# A Real-Time Trajectory Prediction Method of Small-Scale Quadrotors Based on GPS Data and Neural Network

**DOI:** 10.3390/s20247061

**Published:** 2020-12-10

**Authors:** Zhao Yang, Rong Tang, Jie Bao, Jiahuan Lu, Zhijie Zhang

**Affiliations:** National Key Laboratory of Air Traffic Flow Management, College of Civil Aviation, Nanjing University of Aeronautics and Astronautics, Nanjing 210016, China; yangzhao@nuaa.edu.cn (Z.Y.); tangrong258@nuaa.edu.cn (R.T.); lujiahuan@nuaa.edu.cn (J.L.); zhijiez@nuaa.edu.cn (Z.Z.)

**Keywords:** trajectory prediction, quadrotor, gated recurrent unit, position

## Abstract

This paper proposes a real-time trajectory prediction method for quadrotors based on a bidirectional gated recurrent unit model. Historical trajectory data of ten types of quadrotors were obtained. The bidirectional gated recurrent units were constructed and utilized to learn the historic data. The prediction results were compared with the traditional gated recurrent unit method to test its prediction performance. The efficiency of the proposed algorithm was investigated by comparing the training loss and training time. The results over the testing datasets showed that the proposed model produced better prediction results than the baseline models for all scenarios of the testing datasets. It was also found that the proposed model can converge to a stable state faster than the traditional gated recurrent unit model. Moreover, various types of training samples were applied and compared. With the same randomly selected test datasets, the performance of the prediction model can be improved by selecting the historical trajectory samples of the quadrotors close to the weight or volume of the target quadrotor for training. In addition, the performance of stable trajectory samples is significantly better than that with unstable trajectory segments with a frequent change of speed and direction with large angles.

## 1. Introduction

In the recent years, unmanned quadrotors are being widely used in various fields due to its small size, easy manipulation, low cost and high flexibility. The flight of quadrotors may operate with various degrees of autonomy, either under remote control by a human operator or autonomously by onboard computers. With the increasing demand of quadrotors, new requirements are put forward for the current air traffic management system to ensure that numerous unmanned aerial vehicles (UAVs) can operate safely, orderly, and efficiently, especially in the low-attitude airspace of congested urban areas. Among the most important urgent tasks of quadrotor operational management is the real-time prediction of quadrotor trajectory. The accurate trajectory prediction of quadrotors is an important prerequisite in air traffic control automation, as it can provide decision support for UAV conflict detection, the early warning of abnormal behaviors and scientific assessment of airspace condition.

With the development of data mining technology, the trajectory prediction of UAVs has become an increasingly hot research topic. The core function is to predict the short-term flight path of the UAVs in the target airspace through trajectory synthesizer models. As compared with large-scale UAVs, the movement patterns of small-size quadrotors are quite different in terms of the frequent direction change and hovering behaviors, which are more difficult to capture. To this end, this paper aims to: (a) propose a real-time trajectory prediction method for small-size quadrotors based on machining learning techniques; (b) test the performance of the proposed method based on the selection of various training samples; and (c) identify the impact of the frequent change of speed and direction with large angles in prediction accuracy.

The major contributions of this paper can be summarized in three aspects. First, bidirectional gated recurrent unit models were constructed and utilized to learn the historic data, which explicitly consider the information obtained from time-series data with different time intervals. Second, various types of training samples were applied and compared, considering the distribution of performance parameters of quadrotors. Therefore, the impact of selection of training samples on the performance of the proposed model was identified. Third, the influence of unstable trajectory segments with a frequent change of speed and direction with large angles on the performance of the proposed model is examined. The prediction results are compared with those of benchmark methods. The results confirm the superiority of the proposed method in terms of both the prediction accuracy and convergence time.

The rest of the paper is organized as follows. Section 2 presents the related work through a literature review. Section 3 describes the data preparation process. Section 4 discusses the selected methods for real-time trajectory prediction. Section 5 presents the data analysis results and comparison of the predictive performances between the proposed approach and the benchmark models. Section 6 summarizes the conclusions and indicates future research directions.

## 2. Related Work

Previously, some research has been conducted with regard to aircraft trajectory prediction. The methods can generally be categorized into two groups, aircraft performance models based on aerodynamics and data-driven methods. The concept of aircraft performance models is to establish aircraft dynamic and kinematic equations so as to explain the movement pattern [1]. Together with the current state of the aircraft, the subsequent position can be predicted using the established motion equations [2,3,4,5]. The aircraft dynamic and kinematic models used in trajectory prediction are usually based on certain assumptions in order to simplify the mathematical models of aircraft motion. This, in turn, has an impact on the prediction accuracy. As a matter of fact, the factors that affect the aircraft trajectory are very complex, including the computation model, aircraft intent, environmental conditions and performance parameters [6,7,8], etc. While most of relevant parameters are difficult to measure accurately, the usage of aircraft performance models is limited to some ideal conditions due to inaccuracy and instability in real data testing.

In contrast to the aircraft dynamic and kinematic models, the data-driven predicting methods take the trajectory as time series data and consider that the influencing mechanism of various factors on the prediction of aircraft trajectory is implicit in the change of time series data, thus avoiding the requirement for the complete information of various factors, as well as complex motion equations. Machine learning techniques can be used to predict aircraft trajectory, as long as historic information is available, which can be obtained from GPS data [9]. In recent years, an increasing number of studies have been conducted with regard to the implementation of machine learning techniques in predicting the trajectory of moving objects [10,11,12]. By using data mining techniques to extract the active locations, the mobility patterns can be predicted by Markov models [13,14], Kalman filtering [15], Gaussian mixture models [16], support vector machines [17], neural networks [18,19,20,21,22], etc. It has been proved that the prediction results of machine learning techniques are more accurate, reliable and robust in most cases, as compared with traditional methods [23,24].

Although an increasing number of studies are being conducted regarding aircraft trajectory prediction, until recently, however, there has been limited research on the trajectory prediction of unmanned quadrotors. As compared with commercial or fixed wing aircrafts, quadrotors have relatively small sizes, low weights, slow operating speeds, and flexible movement patterns. In some cases, the quadrotors may suddenly change directions or change the dynamics of their motions from flying to hovering. In addition, their operations are at low altitudes, where wind patterns are harder to characterize [25,26]. These factors contribute to the complexity of intent recognition and trajectory prediction. However, most of present literature concentrate on the dynamic modeling and control of unmanned quadrotors [27,28,29]. As a result of these sudden changes in the quadrotor trajectories, the dynamical motion models will have difficulties in maintaining a consistent prediction over long horizons. Research is still needed to be conducted to improve the techniques for quadrotor trajectory prediction for its safe and efficient operation in low altitude airspace.

Recently, the recurrent neural networks (RNN) have been widely recognized as a powerful machine learning tool for the prediction of time-series datasets. Unlike feedforward neural networks, RNNs have feedback loops with recurrent connections between the nodes of the network making them capable of modeling sequences. Noteworthy, long short-term memory (LSTM) and gated recurrent unit (GRU), as variations of RNNs, are designed to capture data dependencies for modeling time series data, which shows a great potential and promise in quadrotor trajectory prediction. It has been recognized that both the LSTM and GRU methods may provide satisfactory results, while the GRU method can outperform LSTM units both in terms of convergence in CPU time and in terms of parameter updates and generalization [30].

Considering the requirement of the real-time prediction of quadrotor location, the prediction accuracy and computation time should be both highlighted and balanced. In this paper, a procedure based on the bidirectional gated recurrent unit (D-GRU) method has been proposed for intention recognition and the short-term trajectory prediction of quadrotors. Historical flight trajectory data of various types of quadrotors are obtained. The D-GRU neural network algorithm is constructed and utilized to learn the historical trajectory data. The prediction results are compared with the traditional GRU method to testify its prediction performance.

## 3. Data Preparation

To meet the research objective, historic trajectory data for various quadrotors were collected. Relevant information was recorded by using airborne GPS sensors, and the trajectory dataset used in the research is collected at intervals of 5 s. The dataset covers 10 kinds of quadrotors, some performance parameters of which are shown in Table 1.

In this table, the second column “Weight” represents the net weight of the quadrotor including rotors. The third column “Max Speed” represents the maximum horizontal speed of the quadrotor in calm wind. The fourth column “Max WS” refers to the maximum wind speed allowed when the quadrotor keeps flying. The last two columns “Max CR” and “Max DR” are the maximum climb rate and descent rate of the quadrotors, respectively.

A total of 924 trajectories were collected, including 61,029 trajectory records. Each record of data includes the coordinated universal time (UTC) time stamp, quadrotor ID, latitude, longitude, altitude, horizontal speed, etc. The numbers of trajectories for each type of quadrotor are shown in the “Trajectory” column in Table 2.

**Figure 1:**
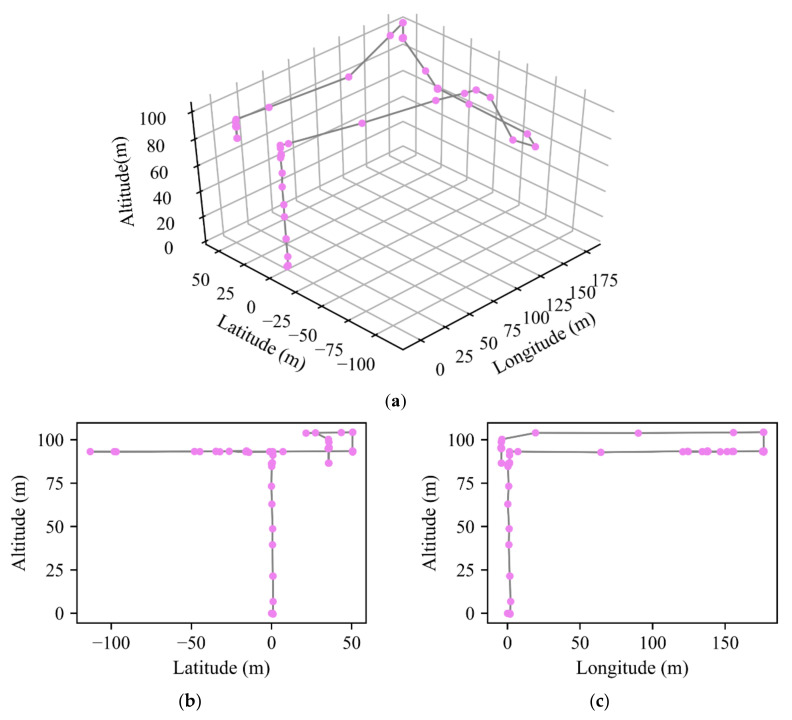
(**a**) The three-dimensional view; (**b**,**c**) the side views of a quadrotor trajectory.

Figure 1 shows a three-dimensional view and two side views of a typical quadrotor trajectory. The figure depicts the frequent and fast changes in the behaviors of the randomly selected quadrotor, such as large-angle direction change, flexible vertical upward and downward movements.

**Figure 2:**
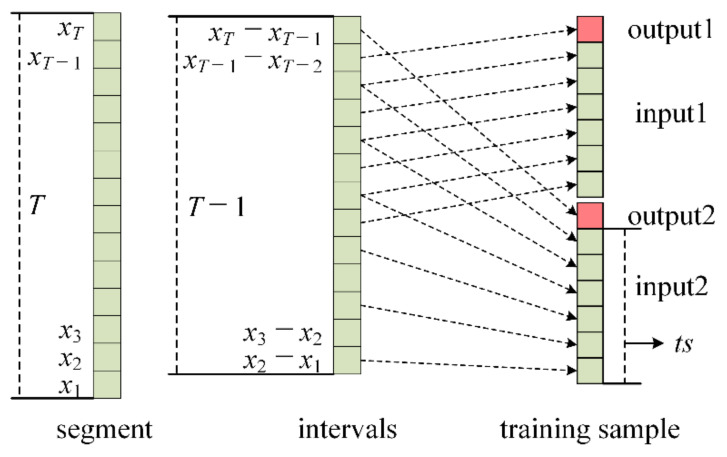
The minimum length of *T* for each segment.

According to the time sequence, the length *ts* required by the trajectory prediction task, each quadrotor trajectory (e.g., the trajectory in Figure 1) is divided into several segments with length *T*. In order to meet the length that is needed for the trajectory prediction, *ts* and *T* satisfy the following relationship, which is also shown in Figure 2:

(1) $$T\geq \left[\left(ts+1\right)\times 2-1\right]+1=\left(ts+1\right)\times 2$$

where (*ts* + 1) is the length of input and output for the trajectory prediction based on every one tracing point; [(*ts* + 1) × 2 − 1] is the length of difference series that is required for trajectory prediction based on every two tracing points as shown in Figure 2; [(*ts* + 1) × 2 − 1] + 1 is the segment length that is required for the computation of the different value of displacement. The number of segments generated by each kind of quadrotor is shown in the “Sample 1” column of Table 2.

According to the performance parameters of the selected quadrotors in Table 1, each segment in Sample 1 is checked, and the segments that meet the maximum performance constraints of the corresponding quadrotor are selected. Since the quadrotors can perform hovering operations, there is no limitation for the maximum turning angular velocity. Therefore, the screening process mainly refers to the limitation in the maximum ground speed ***GS*** of the quadrotor. The normalization of ***GS*** can be expressed as

(2) $$\left|GS\right|={\left|V_{\text{calm}}\right|}^{2}+{\left|WS\right|}^{2}+2\left|V_{\text{calm}}\right|\left|WS\right|\cos\theta$$

where ***V_calm_*** is the speed of the quadrotor in the calm wind; ***WS*** is the wind speed; *θ* is the angle between ***V_calm_*** and ***WS***. The maximum ***GS*** of the quadrotor is generated when ***V_calm_*** is in the same direction as ***WS***, and both reach the maximum critical values, as shown in the “Max Speed” and “Max WS” columns in Table 1. Thus, the maximum displacement of the quadrotor with the maximum ***GS*** can be determined at a unit of time. The segments with displacement per unit of time that exceed the displacement interval in Sample 1 are deleted. The number of segments obtained after screening are shown in “Sample 2” column of Table 2.

**Figure 3:**
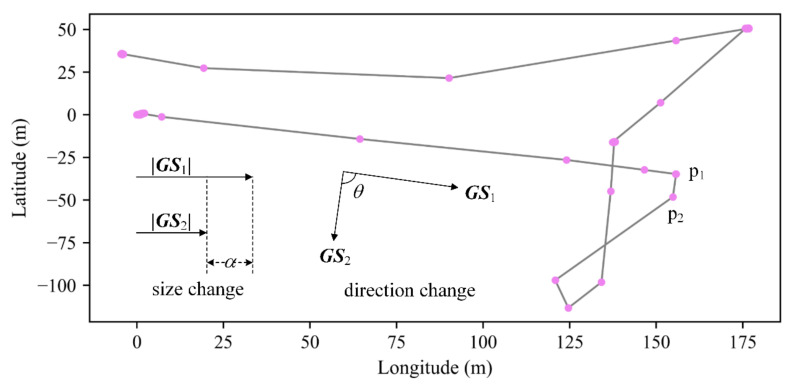
The change in ground speed (***GS***) from the top view.

In order to further demonstrate the distribution of the trajectory data, the variance (*Var*) of the size change of ***GS*** in each segment is calculated, and the frequency (*Freq*) of the direction change of ***GS*** in [0°, 45°], [45°, 90°], [90°, 135°] and [135°, 180°] is counted. The explanation of the size and direction change of ***GS*** is shown in Figure 3, where ***GS***_1_ and ***GS***_2_ are the ground speed of the track points *p*_1_ and *p*_2_, respectively. The direction change of ***GS*** is calculated as the change in displacement direction between the sample point and the previous tracking point.

**Figure 4:**
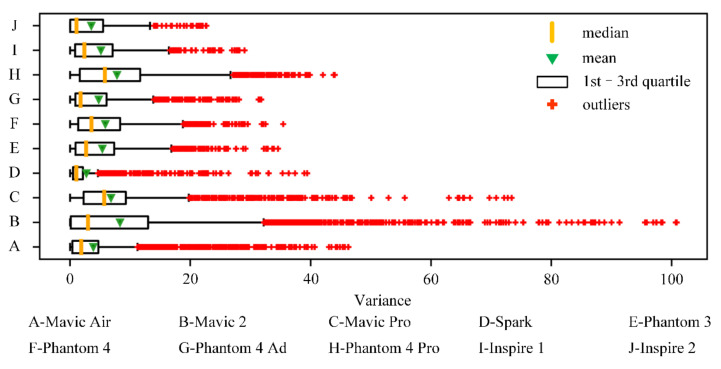
Distribution of the change of ***GS*** for each type of quadrotor.

**Figure 5:**
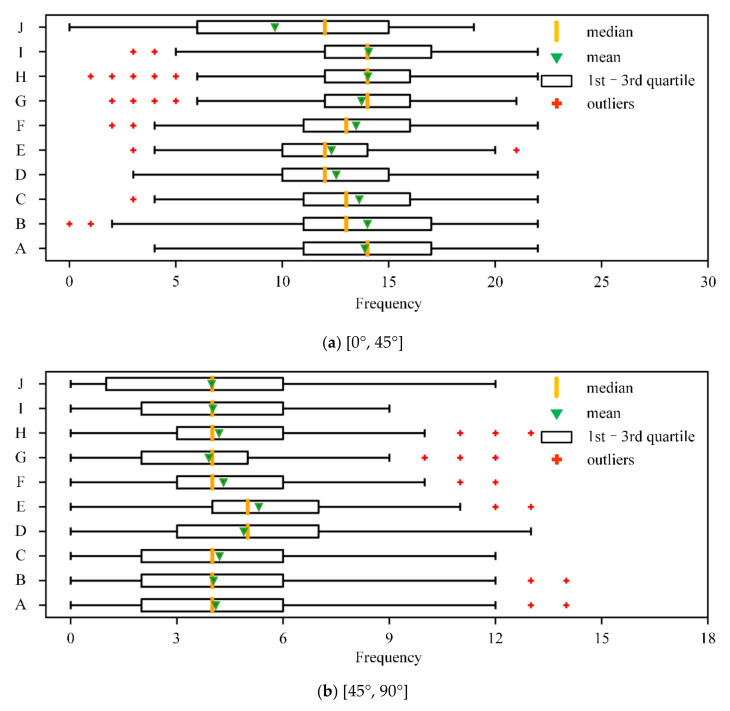

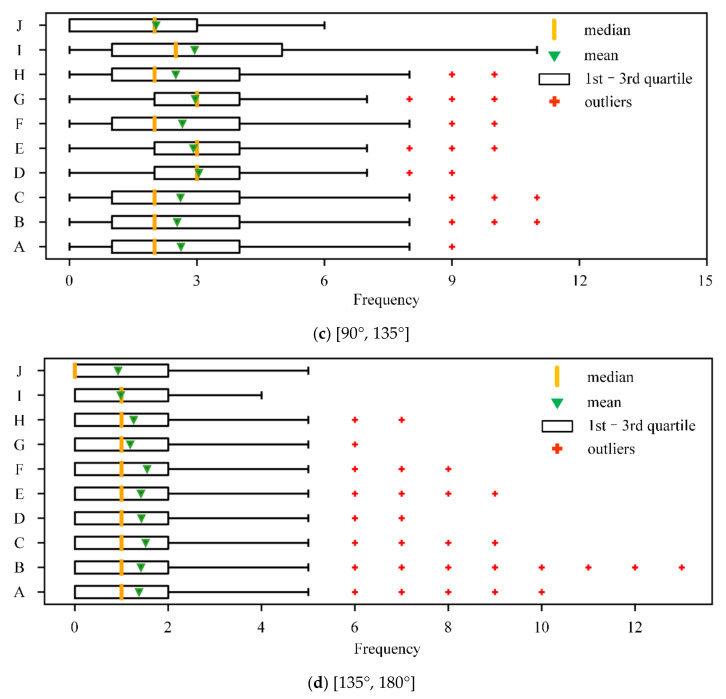
The frequency of the directional change of ***GS*** in the four intervals.

The distribution of *Var* and *Freq* for each type of quadrotor is illustrated in Figure 4 and Figure 5.

According to Figure 4, the distribution of *Var* is quite different for the different types of quadrotors. In Figure 5, the mean value of the *Freq* index generally decreases as the value of the angle interval increases, while the large-angle heading changes still frequently exist during the [135°, 180°] interval, indicating the high flexibility of quadrotors, especially the light quadrotors. Furthermore, the samples of the trajectory segments, which are lower than *Var_max_* and *Freq_max_* for each type of quadrotor, are selected, as shown in the “Sample 3” column of Table 2. *Var_max_* and *Freq_max_* are defined as follows:

(3) $$Var_{max}=max\left(per_{75}{\left(Var_{i}\right)}_{j}\right)$$

(4) $$Freq_{max,k}=max\left(per_{75}{\left(Freq_{i,k}\right)}_{j}\right),k=1,2,3,4$$

where *Var_i_* represents the *Var* of the trajectory segment *i*, and *per*_75_(*Var_i_*)*_j_* represents the upper quartile of the *Var* of the *j*th type of quadrotor. *Freq_i_*_, *k*_ represents the *Freq* of segment *i* in interval *k*; and *per*_75_(*Freq_i_*_,*k*_)*_j_* represents the upper quartile of *Freq* of the *j*th type of quadrotor in interval *k*.

The last column of Table 2 represents the reduction ratio of the number of segments from Sample 2 to Sample 3. It is found that except for the inspire2 quadrotor, the reduction ratio for the other types of quadrotors is over 50%, with 70.03% for Mavic 2 as the highest. The estimated reduction ratios are generally consistent with the variance distribution of changes in ground speed and direction.

## 4. Methodology

In this section, a real-time trajectory prediction method for the quadrotors is constructed based on bidirectional gated recurrent unit (D-GRU) model. The procedure is presented as follows.

### 4.1. The D-GRU Network

**Figure 6:**
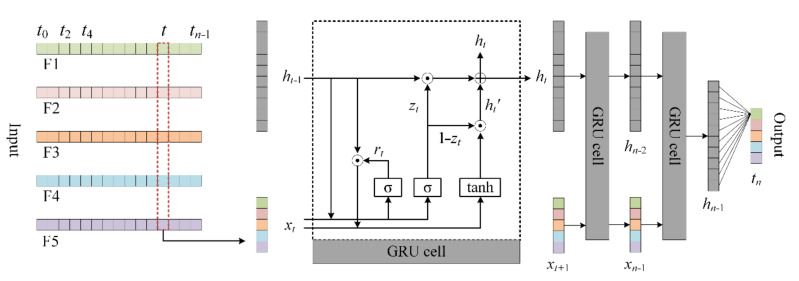
The structure of a single gated recurrent unit (GRU) cell.

Gated recurrent unit (GRU) is an improved recurrent neural network (RNN) proposed by Chung et al. [30]. Similar to long short-term memory (LSTM), GRU is also used to solve the long-term memory problem existing in RNN and the gradient problem in backpropagation. However, in contrast to the structure of the forgetting gate, the input gate and cell gate in LSTM, the GRU network has a reset gate and update gate. The function of the update gate is similar with the combination of the forgotten gate and the input gate in the LSTM, which is responsible for processing the memory and the current input information. In addition, the GRU model has fewer parameters than the LSTM model and requires fewer samples to implement generalization. In the process of the real-time trajectory prediction of quadrotors, historical trajectory samples change rapidly, and the reliable and accurate position prediction by training samples in a short time is of vital importance. Therefore, this paper uses the GRU model to realize the real-time trajectory prediction of small and slow quadrotors. The principle of a GRU unit is shown in Figure 6.

As shown in Figure 6, F1, F2, and F3 represent the change in latitude, longitude, and altitude, respectively. F4 is a change in the size of the ground speed. The change in direction can be reflected as the change of latitude and longitude. F5 is the change in vertical speed. The procedure of the GRU cell is listed as follows.

First, use the sigmoid activation function *σ* to activate the input information of the reset gate so as to obtain the current state *r_t_* of the reset gate. The formula is:

(5) $$r_{t}=\sigma \left(\left[W_{rt}\cdot h_{t-1},U_{rt}\cdot x_{t}\right]\right)$$

where *x_t_* is the input information at the current moment; *h_t_*_−1_ is the hidden state passed at the previous moment; *W_rt_* and *U_rt_* are the weights corresponding to *x_t_* and *h_t_*_−1_, which are updated continuously in training. Similarly, the current state *z_t_* of the update gate can be calculated by the following formula:

(6) $$z_{t}=\sigma \left(\left[W_{zt}\cdot h_{t-1},U_{zt}\cdot x_{t}\right]\right)$$

where *W_zt_* and *U_zt_* are the weights corresponding to *x_t_* and *h_t_*_−1_ in the update gate, respectively.

Second, the reset gate state *r_t_*, the hidden state *h_t_*_−1_, and the input *x_t_* are combined to calculate the memory information *h_t_*′ at the current moment. The formula is:

(7) $$h_{t}^{\prime }=\varphi \left(\left[W_{t}\cdot (r_{t}⊙h_{t-1}),U_{t}\cdot x_{t}\right]\right)$$

where *φ* indicates the tanh activation function; *W_t_* and *U_t_* are the weights corresponding to *x_t_* and reset *h_t_*_−1,_ respectively. When *r_t_* is 0, the memory information at the current time is determined only by the input *x_t_*.

Third, the update gate state *z_t_* is applied to forget and retain the information contained in *h_t_*_−1_ and *h_t_*′, respectively. The formula is:

(8) $$h_{t}=z_{t}⊙h_{t-1}+\left(1-z_{t}\right)⊙h_{t}^{\prime }$$

where *h_t_* is the output of neurons at the current moment and is also the hidden state passed to the next moment.

**Figure 7:**
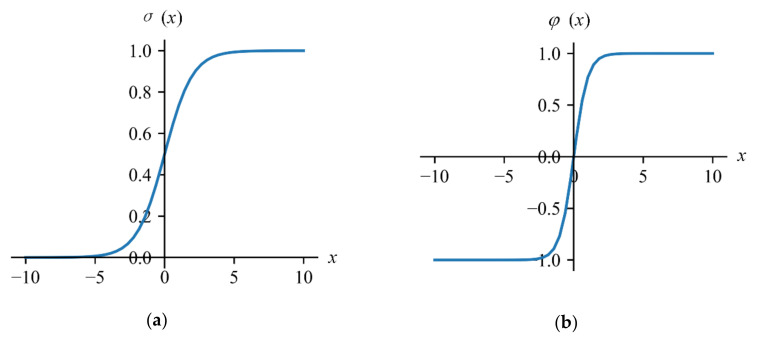
(**a**) The functional curve of the activation function *σ*; and (**b**) the functional curve of activation function *φ*.

In Formulas (5) to (8), the symbol ’·’ means inner product; ‘⊙’ means Hadamard product; *σ* and *φ* are the activation functions for non-linearization. The expressions of them are shown in Formulas (9) and (10), respectively, and the functional curves are shown in Figure 7:

(9) $$\sigma (x)=\frac{1}{1+e^{-x}}$$

(10) $$\varphi (x)=\frac{e^{x}-e^{-x}}{e^{x}+e^{-x}}$$

**Figure 8:**
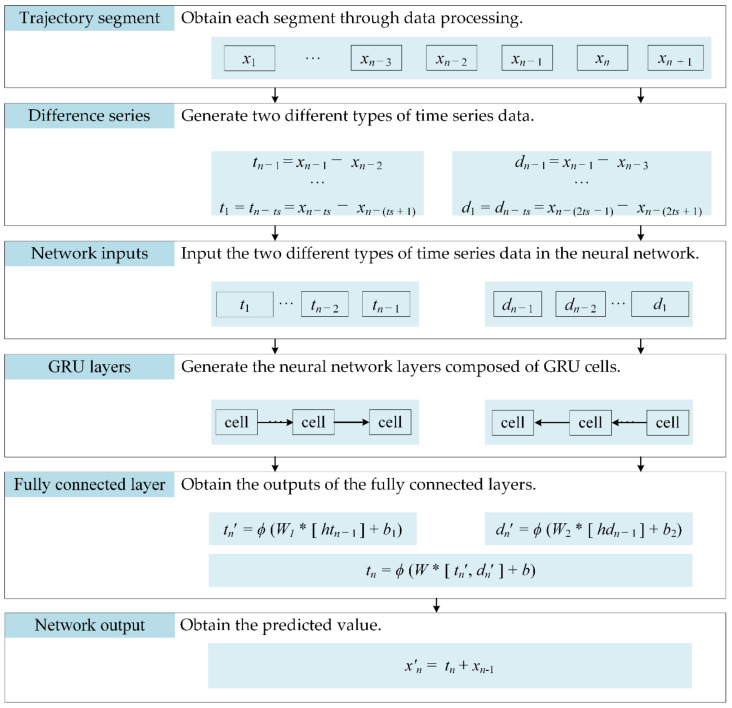
The bidirectional gated recurrent unit (D-GRU) model.

Figure 8 illustrates the structure of the D-GRU model. The location data from different timestamps are used to calculate the change in latitude, longitude and altitude, respectively, during this period. The subsequent change in location is predicted according to the historic movement data. The change in position is calculated for two different intervals, as the left-side of Figure 8 is based on the change in location for two adjacent points, i.e., the historical position data *x_n_*_−1_ and *x_n_*_−2_ in time *n* − 1 and *n* − 2, respectively, are used to predict the change in location *t_n_*_−1_ during time period *n* − 2 to *n* − 1, while the right-side of Figure 8 is based on the change in location at intervals of two points, i.e., the historical position data *x_n_*_−1_ and *x_n_*_−3_ in time *n* − 1 and *n* − 3 are used to predict the change of location *d_n_*_−1_ during the time period from *n* − 3 to *n* − 1. Then, the two outputs, denoted as *t_n_*′ and *d_n_*′, are input into the fully connected layer of the neural network structure. The following steps are provided for the construction of the proposed model.

Step 1: Obtain each segment through data processing, where *x_n_* represents the data at time *n*.
Step 2: According to the requirement of input data length *ts* in the neural network, two different types of time series data are generated, with one- and two-time intervals, respectively.
Step 3: Input the two different types of time series data in the neural network.
Step 4: Generate the neural network layers composed of GRU cells.
Step 5: Obtain the outputs of the fully connected layers. In Figure 8, *ht_n_*_−1_ and *hd_n_*_−1_ represent the hidden states of the GRU layers; *t_n_*′ and *d_n_*′ represent the outputs of the fully connected layers; and *t_n_* is the output of the model.
Step 6: Obtain the predicted value. *x_n_*′ represents the predicted value of the location.

### 4.2. Loss Gunction

In order to suppress the over-fitting of the neural network during training, the L2 regularization method is applied as the loss function on the mean square error for the regression model [31,32], which is a commonly used normalization method in previous research. Based on the L2 normalization method, the optimal value is unique when calculating the gradient, while the L1 normalization method may provide multiple optimal values [33]. The formula is shown as follows:

(11) $$Loss=\frac{1}{n}\sum_{i=1}^{n}{\left(y_{i}^{\prime }-y_{i}\right)}^{2}+\lambda \sum_{k=1}^{K}w_{k}^{2}$$

where *y_i_* and *y_i_*′ are the actual and predicted values of sample *i*, respectively; *λ* is the proportion of regularization in the total loss; and *w* is the trainable weights of the regression model.

### 4.3. Evaluation Metrics

To evaluate the performance of the proposed model, the mean absolute error (*MAE*), root mean squared error (*RMSE*) and mean absolute percentage error (*MAPE*) were calculated for each method, respectively. The equations are shown as follows:

(12) $$MAE=\frac{1}{n}\sum_{i=1}^{n}{\left|y_{i}^{\prime }-y_{i}\right|}^{2}$$

(13) $$RMSE=\sqrt{\frac{1}{n}\sum_{i=1}^{n}{\left(y_{i}^{\prime }-y_{i}\right)}^{2}}$$

(14) $$MAPE=\frac{1}{n}\sum_{i=1}^{n}\left|\frac{y_{i}^{\prime }-y_{i}}{y_{i}}\right|$$

where *y_i_* and *y_i_*′ are the actual and predicted categories of the test sample *i*, respectively, and *n* is the test sample size.

## 5. Test and Results

To testify the impact of training samples on the performance of the proposed algorithm, different subsets selected from the original training samples were applied and the results compared. Taking the Mavic 2 quadrotor as an example, for the objective of the trajectory prediction of this type of quadrotor, the following three subsets of Sample 2 were selected as training samples, and the results are summarized in Table 3:

- Training Sample 2-1: the segments of all types of quadrotors in Sample 2;
- Training Sample 2-2: the segments of Mavic Air, Mavic 2, Mavic Pro and Spark in Sample 2, with weights of less than 1000 g;
- Training Sample 2-3: only the segments of Mavic 2 in Sample 2;
- Training Sample 2-4: half of the sample randomly selected from Sample 2-1.

The four subsets, including Samples 2-1, 2-2, 2-3 and 2-4, were applied as training datasets. Then, the collected historic data for Mavic 2 were input into the trained models to obtain the predicting results.

Similarly, in order to investigate the influence of the unstable trajectory segments, such as a frequent large-angle change in direction and speed, on the performance of the prediction model, the following three subsets of Sample 3 were selected as training samples, and the results are summarized in Table 4:

- Training Sample 3-1: the segments of all types of quadrotors in Sample 3;
- Training Sample 3-2: the segments of Mavic Air, Mavic 2, Mavic Pro and Spark in Sample 3, with weights of less than 1000 g;
- Training Sample 3-3: only the segments of Mavic 2 in Sample 3;
- Training Sample 3-4: half of the sample randomly selected from Sample 3-1.

As shown in Table 3 and Table 4, the following findings can be obtained:

● The proposed D-GRU model produces better prediction results than the GRU model in terms of all the three evaluation indicators. The MAEs of latitude, longitude, and altitude are reduced by at least 11.85, 6.35, and 5.66% in Sample 2, as well as 17.55 9.18, and 4.26% in Sample 3, respectively. Compared with the single-interval sequence GRU model, the D-GRU model can extract more information from the trajectory segment of inherent length *T*, which will try to restrain the irregularity of the transmission pattern learned from the information of single-interval data based on the information of double intervals.
● The prediction accuracies from Samples “2-1” and “3-1” were better than those from Samples “2-4” and “3-4”, indicating that a larger sample dataset for training may help to improve the model performance. The prediction results from Sample “2-2”, Sample “2-3” and Sample “3-2”, Sample “3-3” were significantly better than the prediction results from Sample “2-1” and Sample “3-1”. The MAEs of the optimal prediction results from Sample “2-2” and Sample “2-3” for latitude, longitude, and altitude were reduced by 17.29, 15.25, and 23.86% as compared with Sample “2-1”, and 18.64, 16.44, and 21.97% from Sample “3-2” and Sample “3-3” as compared with Sample “3-1”, respectively. Selecting the historical trajectory samples of the quadrotors close to the weight or volume of the target quadrotor can improve the performance of the prediction model, indicating that the weights and biases learned from the training samples close to the target are more appropriate for trajectory prediction.
● As compared with Sample 2, the prediction results from Sample 3 tends to be much better. Specifically, the MAEs of latitude, longitude, and altitude of the optimal predictions from Sample 3 were reduced by 29.56, 29.21, and 10.00% compared with Sample 2, respectively. This is due to the reason that by introducing the constrains of the *Var* and *Freq* of the trajectory segment, the training trajectory segments in Sample 3 are more stable as compared with Sample 2 in latitude and longitude. By removing unstable trajectory samples such as noise, just like the training sample filtering process, the performance of the trajectory prediction model can be further improved.

**Figure 9:**
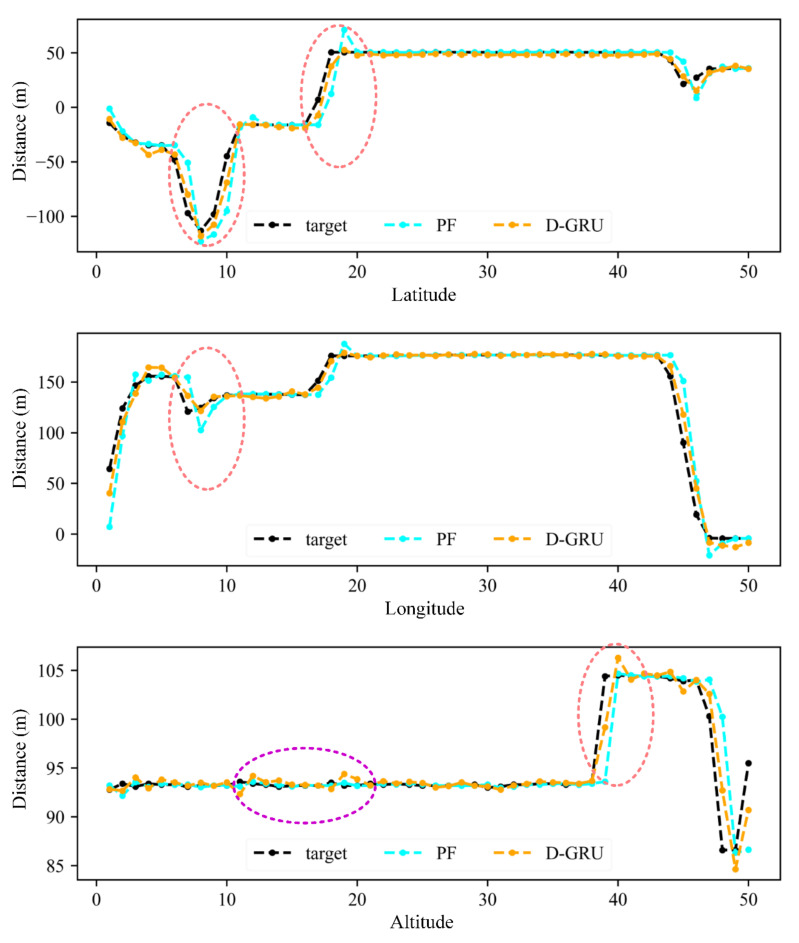
The real-time prediction results using D-GRU and particle filtering (PF) models.

For the objective of real-time prediction, the predicted consecutive trajectory points for the Mavic 2 samples are recorded using the proposed D-GRU models and then compared with the actual location. The traditional particle filtering (PF) model was constructed for comparison as a benchmark method, which was commonly used for trajectory prediction in previous research [34,35,36,37]. The results were summarized in Figure 9 and Table 5. The horizontal axis of Figure 9 represents 50 consecutive points. For the “D-GRU” and “PF” lines, the vertical axis represents the distance from the original longitude, latitude and altitude to the predicted location; and for the target line, the vertical axis represents the distance from the original longitude, latitude and altitude to the actual location.

According to Figure 9 and Table 5, it can be observed that, in general, the prediction accuracy of the proposed D-GRU model is higher than that of the traditional PF model for latitude, longitude and altitude. The D-GRU model especially presents the higher prediction accuracy during the turning points, as depicted in the red circles in Figure 9, while the PF model presents satisfactory prediction results for the stable flight states, as depicted in the violet circle in Figure 9. However, as the PF model is based on kinematic equations, the advantage of the PF model lies in that real-time prediction can be conducted just based on the previous location and speed, while the neural network model requires a historic time-series dataset for training and prediction.

In some cases, the lack of historic trajectories as training samples for the D-GRU model tends to be a potential difficult task. For example, for the case of the invasion of the quadrotor to protected airspace around the airports for the first time, it might be difficult to collect massive datasets for the target quadrotor during a short time period. A solution is that the models can be trained based on the historic trajectory datasets with similar types, weights, or sizes. Then, the real-time surveillance data can be input into the trained models to make prediction. It has been proved above that the prediction accuracy can be improved through the training sample selection.

**Figure 10:**
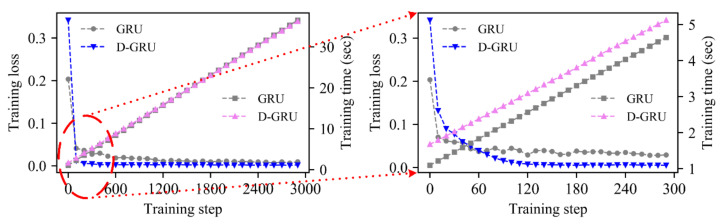
Training loss and training time with respect to the training step.

Otherwise, if large samples of historic trajectory records can be obtained, it is crucial to finish the training and prediction process during a short time period. As the prediction process only takes a very short time, which is only a few milliseconds in our study, most of the prediction time is spent on data training. To further investigate the efficiency of the proposed algorithm, the training loss and training time of the D-GRU and GRU models are recorded and compared, as shown in Figure 10. The CPU type applied in this study is R5-3600, with the frequency of 4.00 GHz and batch size of 100. It can be seen that the training loss of the D-GRU model can converge to a stable state faster than the GRU model from the left sub-picture of Figure 10. The convergence procedure in the first 300 training steps is enlarged in the right of Figure 10. Considering the application of the algorithm in real-time trajectory prediction, the data collection interval is 5 s in this study. The training loss of the D-GRU model is lower than that of the GRU model before the first 5 s according to the right sub-picture of Figure 10, while the training loss of the two models are generally comparable after longer training in the left of Figure 10. The reason for the faster convergence of the D-GRU model is that the GRU model is more prone to overfitting. In order to decrease the training loss steadily, a smaller learning rate should be used. Nevertheless, according to the GRU training loss convergence curve, it is found that there is still a slight overfitting phenomenon.

In addition, it can also be seen from the right side of Figure 10 that although the training convergence speed of the D-GRU model is faster than that of GRU model, it still takes about 3 s to converge to the stable state. It will be difficult to complete the training and prediction process with the current equipment if the required prediction interval is less than 3 s. In order to shorten the training time, the CPU with better computing power may be needed, while this efficiency improvement based on equipment performance often has an upper limit. Therefore, in order to solve the problem of insufficient target historic samples and meet the real-time requirements at the same time, the process of the training sample selection and model pre-training according to the quadrotor type, weight and other characteristics can help to accomplish the real-time prediction task.

## 6. Conclusions

This paper proposed a real-time trajectory prediction algorithm for quadrotors based on D-GRU neural networks. The traditional GRU method is improved by integrating two different prediction frameworks, including the prediction based on the change of location for two adjacent points, as well as the prediction based on the change of location at intervals of two points. The prediction results are compared with the traditional GRU model as the benchmark method. Taking the type of MAVIC2 quadrotor as a case study, the mean position error is 3.10 m for latitude, 3.66 m for longitude and 1.35 m for altitude, respectively. The proposed algorithm improves both the accuracy and efficiency for the short-term trajectory prediction of quadrotors.

Even though the proposed D-GRU approach has exhibited great potential for short-term trajectory prediction, several limitations are still needed to be addressed in future studies. First, this study is focused on the short-term trajectory prediction based on historic location data. As a matter of fact, the real time location is affected by a series of factors such as meteorological parameters such as wind. Future research is still needed to identify the impacts of other significant variables. Second, the paper used the data from several commonly used types of quadrotors as inputs. Data from other types of UAVs can also be applied to further investigate the robustness and applicability of the proposed model, especially those with a different performance of flexibility parameters. The authors recommend that future studies focus on these issues.

## Figures and Tables

**Table 1:** Performance parameters of the selected quadrotors.

Drone Type	Weight (g)	Max Speed (m/s)	Max WS ^a^ (m/s)	Max CR ^b^ (m/s)	Max DR ^c^ (m/s)
Mavic Air	430	19.0	10.7	3.0	2.0
Mavic 2	905	20.0	10.7	5.0	3.0
Mavic Pro	743	18.0	10.7	5.0	3.0
Spark	300	13.9	8.0	3.0	3.0
Phantom 3	1216	16.0	10.7	5.0	3.0
Phantom 4	1380	20.0	10.7	6.0	4.0
Phantom 4 Ad	1368	20.0	10.7	6.0	4.0
Phantom 4 Pro	1388	20.0	10.7	6.0	4.0
Inspire 1	3060	20.0	10.7	5.0	4.0
Inspire 2	3290	26.0	10.7	6.0	4.0

^a^ WS represents wind speed; ^b^ CR represents climb rate; ^c^ DR represents descent rate

**Table 2:** Number of trajectories for each kind of quadrotor.

Type of Quadrotors	Trajectory	Sample 1	Sample 2	Sample 3	Percent
Mavic Air	140	5517	5513	2657	51.80%
Mavic 2	300	14,437	14,384	4311	**70.03%** ^a^
Mavic Pro	185	7725	7581	3471	54.21%
Spark	51	2075	2069	878	57.56%
Phantom 3	64	1966	1792	804	55.13%
Phantom 4	65	2683	2480	999	59.72%
Phantom 4 Ad	18	1241	1172	519	55.72%
Phantom4 Pro	63	4146	3681	1632	55.66%
Inspire 1	26	838	824	824	57.89%
Inspire 2	12	484	403	274	32.01%

^a^ The bold number represents the maximum one.

**Table 3:** Comparison of the prediction accuracy with different training samples for Mavic 2 quadrotors.

Target	Subset	MAE (m) ^a^		RMSE (m) ^b^		MAPE (%) ^c^	
		D-GRU	GRU	D-GRU	GRU	D-GRU	GRU
Latitude	Sample 2-1	5.20	5.32	8.76	10.25	11.13	15.33
	Sample 2-2	**4.40** ^d^	4.98	7.49	8.90	9.64	10.21
	Sample 2-3	4.55	4.74	**7.37**	7.81	**8.45**	11.28
	Sample 2-4	5.37	5.45	9.76	10.98	11.16	15.42
Longitude	Sample 2-1	6.10	6.88	10.86	12.20	10.75	17.59
	Sample 2-2	5.75	6.15	10.72	10.98	**8.95**	10.71
	Sample 2-3	**5.17**	5.52	**9.02**	9.26	9.48	10.86
	Sample 2-4	6.15	7.24	11.78	12.79	11.34	18.26
Altitude	Sample 2-1	1.97	2.46	3.27	3.73	18.09	25.74
	Sample 2-2	1.59	1.59	2.80	3.07	13.54	14.03
	Sample 2-3	**1.50**	1.59	**2.77**	**2.77**	**9.52**	10.17
	Sample 2-4	2.03	2.49	3.33	4.01	18.55	26.74

^a^ MAE is mean absolute error; ^b^ RMSE is root mean squared error; ^c^ MAPE is mean absolute percent error; ^d^ The bold number represents the best prediction result from sample 2-1 to sample 2-4.

**Table 4:** Comparison of the prediction accuracy with different training samples for Mavic 2 quadrotors.

Target	Subset	MAE (m)		RMSE (m)		MAPE (%)	
		D-GRU	GRU	D-GRU	GRU	D-GRU	GRU
Latitude	Sample 3-1	3.81	4.08	6.76	6.80	8.76	10.21
	Sample 3-2	3.59	4.04	6.88	7.00	7.15	9.83
	Sample 3-3	**3.10** ^a^	3.76	**5.88**	6.70	**5.81**	9.48
	Sample 3-4	4.03	4.40	7.01	7.37	9.68	12.59
Longitude	Sample 3-1	4.38	4.34	8.53	8.25	7.57	7.99
	Sample 3-2	3.87	4.52	7.90	8.80	5.89	7.30
	Sample 3-3	**3.66**	4.03	**7.35**	8.27	**5.70**	6.31
	Sample 3-4	4.57	4.73	9.01	9.24	8.19	8.31
Altitude	Sample 3-1	1.73	1.86	3.13	3.41	12.16	12.89
	Sample 3-2	1.46	1.70	2.86	3.39	8.22	8.95
	Sample 3-3	**1.35**	1.41	**2.67**	2.87	**8.11**	8.26
	Sample 3-4	1.91	1.97	3.44	3.38	14.24	13.07

^a^ The bold number represents the best prediction result from sample 3-1 to sample 3-4.

**Table 5:** Comparison of the prediction accuracy between the D-GRU and PF models for Mavic 2 quadrotors.

Target	MAE (m)		RMSE (m)		MAPE (%)	
	D-GRU	PF	D-GRU	PF	D-GRU	PF
Altitude	**1.35** ^a^	1.78	**2.67**	3.99	**8.11**	10.32
Latitude	**3.10**	5.26	**5.88**	9.62	**5.81**	10.07
Longitude	**3.66**	5.35	**7.35**	10.14	**5.70**	8.61

^a^ The bold number represents the better prediction result between D-GRU and PF methods.
